# CONTRAST: a discriminative, phylogeny-free approach to multiple informant *de novo *gene prediction

**DOI:** 10.1186/gb-2007-8-12-r269

**Published:** 2007-12-20

**Authors:** Samuel S Gross, Chuong B Do, Marina Sirota, Serafim Batzoglou

**Affiliations:** 1 Computer Science Department, Stanford University, Stanford, CA, USA; 2 Biomedical Informatics, Stanford University, Stanford, CA, USA

## Abstract

We describe CONTRAST, a gene predictor which directly incorporates information from multiple alignments rather than employing phylogenetic models. This is accomplished through the use of discriminative machine learning techniques, including a novel training algorithm. We use a two-stage approach, in which a set of binary classifiers designed to recognize coding region boundaries is combined with a global model of gene structure. CONTRAST predicts exact coding region structures for 65% more human genes than the previous state-of-the-art method, misses 46% fewer exons and displays comparable gains in specificity.

## Abstract 2

CONTRAST is a gene predictor that directly incorporates information from multiple alignments and uses discriminative machine learning techniques to give large improvements in prediction over previous methods.

## Background

In this work, we consider the task of predicting the locations and structures of the protein-coding genes in a genome. Gene recognition is one of the best-studied problems in computational biology, and as such has been approached through the use of a wide variety of different methods.

Gene recognition methods can be broadly divided into three categories, depending on the type of information they employ. *Ab initio *predictors use only DNA sequence from the genome in which predictions are desired (the *target *genome). Predictors such as GENSCAN [1] and CRAIG [2] fall into this category. *De novo *gene predictors additionally make use of aligned DNA sequence from other genomes (*informant *genomes). Alignments can increase predictive accuracy since protein-coding genes exhibit distinctive patterns of conservation. ROSETTA [3] and CEM [4] were the earliest methods for predicting human genes using alignments. More recent *de novo *gene predictors include TWINSCAN [5], N-SCAN [6], SLAM [7], SGP [8], EvoGene [9], ExoniPhy [10] and DOGFISH [11]. A third class of predictors make use of expression data, usually expressed sequence tag (EST) or cDNA alignments. Pairagon [12], N-SCAN_EST [13], GenomeWise [14] and EXOGEAN [15] belong in this category. These methods can provide highly accurate predictions for genes that are well covered by alignments of expressed sequences. Some programs, such as AUGUSTUS [16], can operate as *ab initio*, *de novo *or expression data-based predictors.

We present CONTRAST (CONditionally TRAined Search for Transcripts), a gene predictor designed primarily for *de novo *prediction but which can also incorporate information from EST alignments. CONTRAST addresses a long-standing problem in *de novo *gene prediction: how to leverage the information contained in multiple informant genomes to achieve predictive accuracy beyond what is possible with any single informant.

The first program to make large gains in human gene prediction performance through the use of an informant genome was TWINSCAN. TWINSCAN was created when human and mouse were the only sequenced vertebrates, and was therefore designed to use only one informant species at a time. As more genomes became available, there was a strong expectation that the additional information provided by deeper alignments would lead to further improvements in accuracy. Several predictors able to use multiple informants, such as ExoniPhy and EvoGene, were created. These programs performed better when they had access to several informants rather than just one, but they were not able to outperform TWINSCAN on genome-wide tests of accuracy. N-SCAN was the first *de novo *predictor to achieve a higher level of performance on human than TWINSCAN. However, despite being designed expressly for the purpose of incorporating information from multiple informants, N-SCAN performs as well using mouse as its only informant as it does with any combination of informant genomes [6].

Until now, no *de novo *gene predictor had been able to exceed N-SCAN's single informant performance [17]. We show that CONTRAST achieves a substantial improvement over state-of-the-art performance in *de novo *gene prediction, and furthermore that this improvement is in large part a result of CONTRAST's ability to effectively make use of multiple informants.

## Results

### Overview of CONTRAST

CONTRAST consists of two main components. The first is a set of classifiers designed to recognize the boundaries of coding regions (start and stop codons and splice sites) based on local information contained in a small window around a potential boundary. The second is a global model of gene structure that integrates output from the classifiers with additional features of a multiple alignment to predict complete genes. We adopt this two-stage approach because it greatly simplifies the task of learning parameters from training data. Training the boundary classifiers requires only short alignment windows corresponding to positive or negative examples of a specific type of coding region boundary. Thus, feature-rich classifiers can be trained efficiently in isolation. The global model can then be trained on the full set of training data, treating the classifiers as black boxes. This avoids the need for the global model to incorporate the large number of features required for accurate recognition of coding region boundaries. We use discriminative machine learning techniques (support vector machines (SVMs) [18] and a conditional random field (CRF) [19]) for both components of CONTRAST, rather than generative models (for example, phylo-hidden Markov models [20]) used by previous *de novo *predictors. This allows CONTRAST to avoid modeling the complex evolutionary process reflected in a multiple alignment and instead concentrate on using information in the alignment to produce more accurate predictions.

### Human gene prediction

To test the accuracy of CONTRAST, we generated predictions for the entire March 2006 build of the human genome (NCBI build 36.1/UCSC hg18). We used the February 2007 consensus coding sequence (CCDS) annotations [21] as our set of known genes. This set contained 16,008 genes and 18,290 transcripts. A four-fold cross-validation procedure was used to estimate how well CONTRAST predicts genes not present in its training set. The genomic alignments we used came from a MULTIZ [22] multiple alignment of 16 vertebrate species. We used 11 informants from the alignment: macaque (rheMac2), mouse (mm8), rat (rn4), rabbit (oryCun1), dog (canFam2), cow (bosTau2), armadillo (dasNov1), elephant (loxAfr1), tenrec (echTel1), opossum (monDom4) and chicken (galGal2).

We discarded alignments from the chimpanzee genome as well as the frog, zebrafish, fugu and tetraodon genomes because of their very small or very large evolutionary distances from human (previous work indicates that non-primate mammals tend to make the most effective informants for human [23]). All data was downloaded from the UCSC genome browser [24]. The genome was masked using RepeatMasker [25] with low-complexity masking disabled.

To determine how much CONTRAST benefits from the availability of multiple informants, we also ran 11 additional sets of predictions, each one using a single informant. We found the most effective single informant to be mouse, consistent with results for other gene predictors [6,23].

For comparison, we evaluated the accuracy of N-SCAN predictions for the same build of the human genome. The N-SCAN predictions were downloaded from the UCSC genome browser and used mouse as the only informant. These predictions represent the best results for N-SCAN; no combination of additional informants has been found to significantly improve N-SCAN's accuracy (Michael R Brent and Jeltje van Baren, personal communication). All evaluations were performed using the Eval package [26]. Table 1 shows the accuracy of CONTRAST using all 11 informants, its accuracy using mouse alone and the accuracy of N-SCAN. As CONTRAST only predicts the protein-coding portions of a gene, we ignored untranslated regions when measuring performance.

**Table 1:** De novo gene prediction performance for human. Sensitivity (Sn) and specificity (Sp) were evaluated at the gene, exon and nucleotide levels and reported as percentages. Also shown are the average number of genes and exons predicted for each cross-validation fold. The column headings indicate the predictor and informants used.

	N-SCAN (mouse)	CONTRAST (mouse)	CONTRAST (11 informants)
Gene Sn	35.6	50.8	58.6
Gene Sp	25.1	29.3	35.5
Exon Sn	84.2	90.8	92.8
Exon Sp	64.6	70.5	72.5
Nucleotide Sn	90.8	96.0	96.9
Nucleotide Sp	67.9	70.0	72.0
Genes predicted	22,596	27,614	26,260
Exons predicted	196,643	211,431	210,180

Sensitivity and specificity were evaluated at the gene, exon and nucleotide levels. Sensitivity was calculated by dividing the number of correctly predicted genes, exons or nucleotides by the total number in the evaluation set, while specificity was calculated by dividing the number of correct predictions by the total number of predictions. Exon predictions were only counted as correct if they matched the boundaries of an exon in the evaluation set exactly; gene predictions were counted as correct if they matched any transcript in the evaluation set exactly. Note that the specificity numbers we report are underestimates, because any predictions not found in the set of known genes were counted as incorrect.

CONTRAST shows a marked improvement over N-SCAN at all three levels of evaluation when using mouse as its only informant. When the other informants are added, CONTRAST's accuracy rises considerably. Using 11 informants, CONTRAST is able to correctly predict an exact coding region structure for over half of all genes, and generates a correct prediction for more than nine out of ten exons. This represents a 65% increase in gene sensitivity and a 46% reduction in exon error rate over the previous state of the art, with similar improvements in specificity. Table 2 shows a breakdown of exon-level accuracy. CONTRAST was both more sensitive and more specific than N-SCAN for each of the four exon types. N-SCAN's exon overlap sensitivity was 91.2%, meaning 8.8% of the exons in the evaluation set did not overlap any of its predicted exons. CONTRAST achieved an exon overlap sensitivity of 96.9%, identifying thousands of exons missed completely by N-SCAN.

**Table 2:** Breakdown of exon-level accuracy for human. The first two rows show sensitivity and specificity for all exons when a prediction is counted as correct if it overlaps an exon in the evaluation set by at least 1 bp. The remaining rows show sensitivity and specificity for the exact prediction of the four different exon types: initial, internal, and terminal exons in multi-exon genes, and exons that contain a gene's full coding region.

	N-SCAN (mouse)	CONTRAST (mouse)	CONTRAST (11 informants)
Exon overlap Sn	91.2	95.5	96.9
Exon overlap Sp	69.6	73.9	75.5
Initial exon Sn	60.9	72.9	76.9
Initial exon Sp	48.8	54.1	56.9
Internal exon Sn	89.4	94.6	96.2
Internal exon Sp	68.7	76.1	77.4
Terminal exon Sn	70.4	80.6	83.5
Terminal exon Sp	53.8	59.8	61.9
Single exon Sn	45.9	65.2	67.5
Single exon Sp	27.7	18.8	24.8

### Effect of the informant set on accuracy

From the results of the previous section, it is clear that CONTRAST's accuracy is improved significantly by the availability of multiple informants. We performed an experiment to quantify the gain in performance as more informants are added. Specifically, we tested how the accuracy of CONTRAST's coding boundary classifiers depends on the choice of informants. We started with classifiers that used only human sequence and added informants one at a time. At each stage, we added the informant that led to the largest relative reduction in error rate, averaged over the classifiers. We measured a classifier's error rate as the fraction of misclassified examples from an evaluation set with an equal number of positive and negative examples. See the materials and methods section for a description of how the training and evaluation examples were obtained.

**Figure 1:**
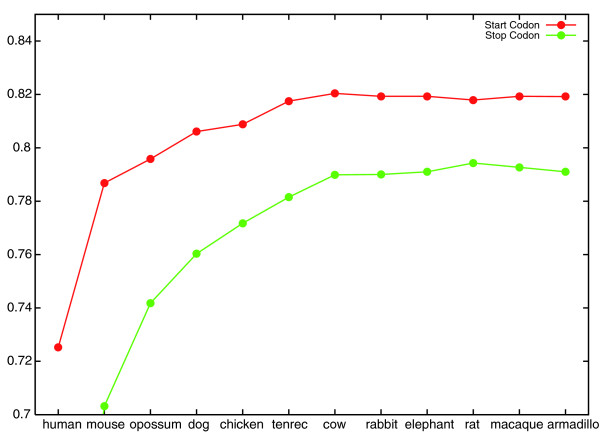
**Start and stop codon classifier accuracy increases as informants are added**. The graph shows the generalization accuracy of CONTRAST's start and stop codon classifiers as more informants are added. The *x*-axis labels indicate the most recently added informant. For example, at the point labeled 'chicken', the informant set consists of mouse, opossum, dog and chicken.

**Figure 2:**
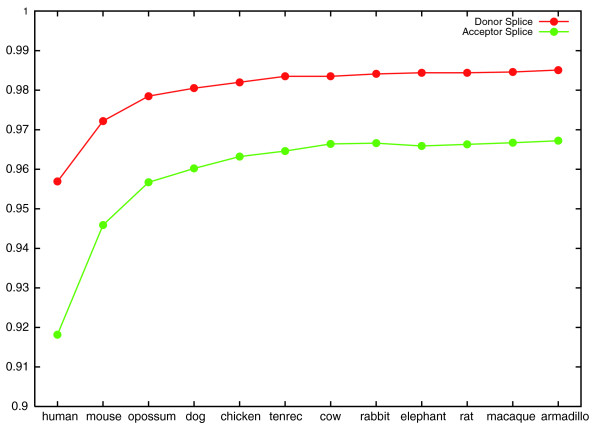
**Splice site classifier accuracy increases as informants are added**. The graph shows the generalization accuracy of CONTRAST's donor and acceptor splice site classifiers as more informants are added. The *x*-axis labels indicate the most recently added informant. For example, at the point labeled 'chicken', the informant set consists of mouse, opossum, dog and chicken.

As the training data included few examples of donor sites with a 'GC' consensus, the error rate of the GC donor site classifier was subject to large fluctuations and we excluded it from consideration. The results of this experiment are shown in Figures 1 and 2. Improvements in classifier accuracy continued as mouse, opossum, dog, chicken, tenrec and cow were added, after which little or no improvement was observed. We note that each of the species added after these six is either only sequenced to low coverage (rabbit, elephant, armadillo), very similar to a species already included (rat) or very similar to the target (macaque). It is an open question whether the availability of more informant genomes would further improve CONTRAST's performance.

The above greedy procedure for selecting informants requires approximately *N*^2 ^experiments, where *N *is the number of possible informants. As training the global gene model is far more expensive than classifier training, it was not practical to perform a similar test using gene prediction accuracy, rather than classifier accuracy, as a metric.

### Prediction with ESTs

We also tested CONTRAST's ability to incorporate data from EST alignments. For this experiment, we used BLAT [27] alignments of all human ESTs in GenBank [28] to the human genome, obtained from the UCSC genome browser. We created predictions using EST information along with alignments from either mouse alone or all 11 informants. We compared the accuracy of these predictions with those from N-SCAN_EST, a version of N-SCAN that makes use of EST alignments. Table 3 shows the results. Both predictors perform significantly better in these tests when EST information is used. However, the results of the experiments with EST data should be interpreted with caution. Nearly all of the known human genes used for evaluation have been discovered by randomly sequencing cDNA libraries, yet it appears that many human genes cannot be found this way [29,30]. Thus, it is likely that cross-validation experiments using the current set of known human genes overestimate the performance of predictors that consider EST evidence. This effect is even more pronounced for predictors that make use of full-length cDNA sequences. Both CONTRAST and N-SCAN_EST use EST evidence to supplement information from genomic alignments in a similar way, allowing for a fair comparison between the two methods. However, we did not compare CONTRAST to programs or pipelines that rely heavily on expressed sequence data.

**Table 3:** Performance for human using EST evidence. Sensitivity (Sn) and specificity (Sp) were evaluated at the gene, exon and nucleotide levels and reported as percentages. Also shown are the average number of genes and exons predicted for each cross-validation fold. The column headings indicate the predictor and informants used.

	N-SCAN_EST (mouse + ESTs)	CONTRAST (mouse + ESTs)	CONTRAST (11 informants + ESTs)
Gene Sn	46.8	60.7	65.4
Gene Sp	31.7	40.6	46.2
Exon Sn	89.7	92.6	93.9
Exon Sp	66.9	74.8	76.2
Nucleotide Sn	93.7	95.7	96.7
Nucleotide Sp	69.3	74.3	75.8
Genes predicted	23,339	23,787	22,507
Exons predicted	202,111	203,253	202,342

### Prediction on the EGASP test set

EGASP was a recent community experiment designed to evaluate the state-of-the-art in human gene prediction accuracy [17]. Researchers submitted sets of predictions for 33 of the 44 ENCODE regions, which span approximately 1% of the human genome and were subject to an intensive annotation effort. To compare the accuracy of CONTRAST with the predictors evaluated in EGASP, we trained CONTRAST on the 99% of the human genome not included in an ENCODE region. We used the May 2004 build of the human genome (NCBI35/UCSC hg17), because this was the latest build available at the time of the EGASP experiment. We used a MUTLIZ alignment of the same 17 species as the 17-way alignment described above. No EST information was used. We generated predictions for the ENCODE regions and sent them to one of the EGASP organizers for evaluation (Paul Flicek, personal communication).

The results of the evaluation, along with results for the 19 predictors entered in EGASP, are shown in Table 4. EGASP divided predictors into four categories based on what type of information they were allowed to use as input: Category 1 (any information), Category 2 (the human genome sequence only, that is, *ab initio *predictors), Category 3 (protein, mRNA, or EST evidence and genomic alignments) and Category 4 (genomic alignments only, that is, *de novo *predictors). As many genes in the ENCODE regions were well annotated prior to EGASP, predictors of Categories 1 and 3 were at a substantial advantage. For example, aligning RefSeq mRNAs to the genome with BLAT produces a set of predictions that is quite accurate (see Table 4).

**Table 4:** Performance on the EGASP test regions. Sensitivity (Sn) and specificity (Sp) are shown at the nucleotide (Nuc), exon, transcript (Trans) and gene levels for CONTRAST and the 19 predictors entered in the EGASP experiment. At each level of evaluation, the performance of the predictor with the highest average sensitivity and specificity for a given category is shown in bold. Also shown is the performance of the UCSC RefGene track, which consists of RefSeq cDNAs aligned to the genome. The track was evaluated just before the start of the EGASP workshop.

	Nuc Sn	Nuc Sp	Exon Sn	Exon Sp	Trans Sn	Trans Sp	Gene Sn	Gene Sp
RefGene Track	85.34	98.50	73.23	94.67	41.91	75.21	77.03	82.56
								
Category 1 (any information)								
AUGUSTUS-any	94.42	82.43	74.67	76.76	22.65	35.59	47.97	35.59
FGENESH++	91.09	76.89	75.18	69.31	36.21	41.61	69.93	42.09
JIGSAW	**94.56**	**92.19**	**80.61**	**89.33**	**34.05**	**65.95**	**72.64**	**65.95**
PAIRAGON-any	87.77	92.78	76.85	88.91	39.29	60.34	69.59	61.32
								
Category 2 (target sequence)								
AUGUSTUS-abinit	**78.65**	**75.29**	**52.39**	**62.93**	**11.09**	**17.22**	**24.32**	**17.22**
GENEMARK.hmm-A	78.43	37.97	50.58	29.01	6.93	3.24	15.20	3.24
GENEMARK.hmm-B	76.09	62.94	48.15	47.25	7.70	7.91	16.89	7.91
GENEZILLA	87.56	50.93	62.08	50.25	9.09	8.84	19.59	8.84
								
Category 3 (protein, mRNA, EST)								
ACEVIEW	90.94	79.14	85.75	56.98	44.68	19.31	63.51	48.65
AUGUSTUS-EST	92.62	83.45	74.10	77.40	22.50	37.01	47.64	37.01
ENSEMBL	90.18	92.02	77.53	82.65	**39.75**	**54.64**	71.62	67.32
EXOGEAN	84.18	94.33	79.34	83.45	42.53	52.44	63.18	80.82
EXONHUNTER	90.46	59.67	64.44	41.77	10.48	6.33	21.96	6.33
PAIRAGON+NSCAN_EST	**87.56**	**92.77**	**76.63**	**88.95**	39.29	60.64	**69.59**	**61.71**
								
Category 4 (genomic alignments)								
CONTRAST	**94.44**	**89.25**	**77.68**	**86.02**	**25.12**	**47.87**	**53.04**	**47.87**
AUGUSTUS-dual	88.86	80.15	63.06	69.14	12.33	18.64	26.01	18.64
DOGFISH	64.81	88.24	53.11	77.34	5.08	14.61	10.81	14.61
MARS	84.25	74.13	65.56	61.65	15.87	15.11	33.45	24.94
NSCAN	85.38	89.02	67.66	82.05	16.95	36.71	35.47	36.71
SAGA	52.54	81.39	38.82	50.73	2.16	3.44	4.39	3.44

CONTRAST's accuracy was significantly higher than the other predictors in Category 4 at all levels of evaluation. The average of CONTRAST's nucleotide sensitivity and specificity was higher than any other predictor (regardless of category) except for JIGSAW, an ensemble method that combines the output of other predictors. CONTRAST's average exon level performance was exceeded only by JIGSAW and the two predictors making use of PAIRAGON, a system for aligning cDNAs to a genome. However, at the transcript and gene levels, predictors that used expression data tended to show better performance than CONTRAST.

The difference in CONTRAST's performance on the CCDS test set and the EGASP test set are a consequence of the very different compositions of the two sets. The CCDS set is known to be fairly incomplete, containing only 16,008 genes of an estimated 20,000-25,000. Nearly 90% of the genes in the CCDS set have only one associated transcript, with an average of 1.14 transcripts per gene. The GENCODE annotations used a gold standard for EGASP so are believed to be much more complete, and this idea is supported by the higher specificities observed for the EGASP test set. Furthermore, alternative splice forms are very prevalent in the EGASP set, with 2.19 transcripts per gene on average. As CONTRAST does not predict alternative splicing if two exon annotations overlap, it is able to make a correct prediction for at most one of the two. This may explain why CONTRAST's exon sensitivity was measured at only 77.7% with respect to the EGASP test set, which contained many overlapping exons, but well over 90% with respect to the CCDS test set, which contained few. This explanation would require CONTRAST to preferentially predict splice forms present in the CCDS annotation over those not present. We speculate that splice forms in the CCDS set may have characteristic properties that set them apart from alternative splice forms, such as a higher degree of conservation or stronger splice site signals.

### Drosophila melanogaster gene prediction

To test how well CONTRAST performs on genomes distant from human, we generated predictions for the April 2004 assembly of the *Drosophila melanogaster *genome (BDGP Release 4/UCSC dm2). We used UCSC alignments of RefSeq mRNAs to the *Drosophila melanogaster *genome as our set of known genes. The set was filtered to remove annotations likely to contain errors. In particular, we discarded annotations containing in-frame stop codons, coding regions with a length not divisible by three or splice sites not matching an established consensus ('GT', 'GC' or 'AT' for donor sites, 'AG' or 'AC' for acceptor sites). After filtering, the known gene set contained 10,891 genes and 16,604 transcripts. The genomic alignments we used came from a MULTIZ multiple alignment of 12 *Drosophila *species and 3 additional insects (mosquito, honeybee and red flour beetle). We used all 13 informants from the alignment: *Drosophila simulans *(droSim1), *Drosophila sechellia *(droSec1), *Drosophila yakuba *(droYak2), *Drosophila erecta *(droEre2), *Drosophila ananassae *(droAna3), *Drosophila pseudoobscura *(dp4), *Drosophila persimilis *(droPer1), *Drosophila willistoni *(droWil1), *Drosophila virilis *(droVir3), *Drosophila mojavensis *(droMoj3), *Drosophila grimshawi *(droGri2), *Anopheles gambiae *(anoGam1), *Apis mellifera *(apiMel2) and *Tribolium castaneum *(triCas2).

We again compared CONTRAST with N-SCAN, the most accurate previous system for *de novo *prediction in *Drosophila melanogaster *[6]. We used a four-fold cross-validation procedure as in the previous experiments. Table 5 shows the accuracy of CONTRAST using all 13 informants, its accuracy using the best single informant (*Drosophila ananassae*) and the accuracy of N-SCAN using *Drosophila yakuba*, *Drosophila pseudoobscura *and *A. gambiae *as informants. The N-SCAN predictions we used were taken from the UCSC genome browser and represent the most accurate result for N-SCAN (Randall Brown and Michael Brent, personal communication). In contrast to the case for human gene prediction, the availability of multiple informants increases N-SCAN's accuracy on *Drosophila melanogaster*. However, CONTRAST performs better even when using only one informant. Furthermore, its accuracy improves significantly with the addition of the other 12 informants.

**Table 5:** De novo gene prediction performance for Drosophila melanogaster. Sensitivity (Sn) and specificity (Sp) were evaluated at the gene, exon and nucleotide levels and reported as percentages. Also shown are the average number of genes and exons predicted for each cross-validation fold. The column headings indicate the predictor and informants used.

	N-SCAN (3 informants)	CONTRAST (*Drosophila ananassae*)	CONTRAST (13 informants)
Gene Sn	59.7	63.1	66.1
Gene Sp	46.4	48.3	52.7
Exon Sn	79.8	81.2	82.4
Exon Sp	67.9	71.6	74.2
Nucleotide Sn	96.2	96.3	96.9
Nucleotide Sp	79.4	82.4	83.4

### Availability of predictions and software

Gene predictions for the human and *Drosophila melanogaster *genomes, as well as source code for our implementation of CONTRAST, are available on the CONTRAST web site [31]. We are currently in the process of generating predictions for many other genomes, which will appear on the web site and as custom tracks for the UCSC genome browser.

## Discussion

### Evaluating gene predictors

It is important to remember that comparing the performance of gene predictors is not a completely straightforward proposition. When evaluating a predictor, we are primarily interested in estimating how accurately it can identify unknown genes. In practice, this is usually accomplished by training the predictor on a portion of a set of known genes and then evaluating its performance on the remainder of the set. We can trust such an estimate as far as we are willing to assume that the predictor is as good at predicting unknown genes as it is at predicting known genes. This assumption is violated for predictors based on expression data, because we expect unannotated genes to be associated with fewer and lower-quality expressed sequences than known genes. It is also reasonable to expect that in fairly well-annotated organisms such as human and *Drosophila melanogaster*, unannotated genes may differ on average from annotated genes in terms of properties such as degree of conservation, length and number of exons.

In addition, the choice of annotation set to use for training and evaluation can have a significant effect on the measured accuracy of a predictor, as evidenced by the differences in CONTRAST's performance on the EGASP and CCDS sets. In particular, it is becoming clear that alternative splicing is extremely prevalent in mammalian genomes, yet annotation sets such as RefSeq and CCDS typically contain only one transcript for most genes. The results we have presented should be interpreted with these considerations in mind.

### Design philosophy

In this work we have focused mainly on *de novo gene *recognition. Accurately predicting the structure of a gene in a *de novo *manner (that is, from genomic sequence alone) is a much more difficult problem than making a prediction based on expressed sequences. For example, if a high-quality, full-length cDNA is available for a particular gene, it is a relatively simple matter to align the cDNA back to the genome and thus recover the exon-intron structure of one of the gene's transcripts. In the absence of a full-length cDNA, genes well covered by EST alignments can be predicted accurately by merging overlapping ESTs to form a complete gene structure. The vast majority of known genes have been discovered by randomly sequencing cDNA libraries to obtain a large number of expressed sequences and then applying expression-based prediction methods. However, this approach has its limits. Genes expressed at low levels or with highly restricted patterns of expression may escape even a very deep level of sequencing. This appears to be the case for a significant fraction of human and mouse genes: results from the Mammalian Gene Collection project show that random sequencing methods reach saturation long before a complete set of genes can be recovered [29]. For genes whose full structure cannot be determined based on expression evidence alone, we must rely on other methods to complete the annotation. The most promising option is the targeted experimental validation of computational predictions using RT-PCR [32-34]. These predictions need not be purely *de novo*, as incorporating EST alignments can help guide predictions on genes with partial EST coverage. However, the accuracy of the predictions on genes or parts of genes not covered by ESTs will depend on our ability to recognize gene structures from genomic sequence. These considerations explain the design philosophy behind CONTRAST, which was intended to be a highly accurate *de novo *predictor with the additional capability of incorporating EST evidence when available. Large-scale projects aiming to identify new genes through the experimental confirmation of *de novo *predictions have already met with considerable success. For example, thousands of novel human exons predicted by N-SCAN and not covered by any EST alignments have been verified by RT-PCR experiments (Computational prediction and experimental validation of novel human genes for the mammalian gene collection, Siepel et al, in preparation). Such projects will benefit greatly from increases in the accuracy of *de novo *gene prediction.

### Discriminative approach

As we have shown, CONTRAST achieves a substantial increase in performance over previous *de novo *predictors in the human and *Drosophila melanogaster *genomes. The fact that CONTRAST performs well on both of these distantly related species suggests that similar results will hold for a variety of higher eukaryotes. We believe a key factor in CONTRAST's success is the unique way in which it uses alignment information to make predictions. Previous predictors (for example, N-SCAN, ExoniPhy, EvoGene and DOGFISH) have integrated information from multiple alignments through the use of evolutionary models that define a probability distribution over alignment columns. These models use independence assumptions derived from a phylogenetic tree to factor the high-dimensional joint distribution over all of the characters in a column into a product of distributions involving at most two characters each. To facilitate this, hidden variables corresponding to the value of ancestral characters are introduced. Gene predictors that use multiple alignments typically contain many of these models, each one corresponding to a particular annotation of the target species character (for example, coding, intronic or intergenic). The use of evolutionary models has been necessitated by the fact that previous gene predictors have been based on some variation of a hidden Markov model (HMM). HMMs are generative models, meaning they define a joint distribution over their input data and a labeling of that data. Thus, HMM-based gene predictors are obligated to model multiple alignments explicitly if they wish to use them as input.

Instead of learning to model the properties of different types of alignment columns and then using these models to predict genes, CONTRAST attempts to learn parameters that approximately maximize the accuracy of its boundary classifiers and global gene model. This type of approach is known as *discriminative*. Discriminative methods have been shown to outperform generative approaches in a wide variety of settings [35-39]. CONTRAST uses SVMs for its coding boundary classifiers and a CRF for its global model of gene structure. Both models directly use features of the multiple alignment without postulating hidden ancestral characters or making assumptions about evolutionary relationships.

### Relationship to previous work

A first attempt to use CRFs for gene prediction was described in [40]. The authors show that a CRF-based gene predictor outperforms a HMM-based predictor, GENIE [41], when both predictors use protein alignments. However, GENIE performs better in *de novo *prediction experiments and no comparisons with more accurate *de novo *predictors such as N-SCAN were made.

Two more recent studies have also addressed the issue of using discriminative methods for *de novo *gene prediction. In the first study, the authors introduce CRAIG, an *ab initio *gene predictor based on a semi-Markov CRF [2]. CRAIG does not make use of alignment information, but instead uses a rich set of target sequence features. CRAIG reaches a significantly higher level of accuracy than other *ab initio *predictors, which are based on generative models. Although its overall performance is not on a par with the best *de novo *predictors [2,17], the fact that CRAIG makes predictions without using alignments means it is well suited for discovering rapidly evolving genes. The second study describes Conrad [42], an alignment-based gene predictor that takes a partially discriminative approach. Conrad uses a semi-Markov CRF to combine output from generative evolutionary models with non-probabilistic features based on alignment gap patterns and EST evidence. Conrad has been shown to outperform TWINSCAN on a fungal genome, but has not been applied to the more difficult task of predicting genes in a large genome with long introns. We were not able to test Conrad on the human or *Drosophila melanogaster *genomes as its current implementation does not support the parallelization of training computations across a compute cluster, making training on large genomes prohibitively expensive [43].

The two-stage approach used in CONTRAST, in which coding region boundary classifiers are separated from a global model of gene structure, is similar to the strategy employed by DOGFISH. There are three principal differences between the two methods. First, the classifiers used by DOGFISH consider a much larger window around potential coding boundaries, including up to 100 positions in the coding region. In CONTRAST, at most six coding positions are considered by a classifier; most of the task of scoring coding sequence is left to the global model. Second, DOGFISH's classifiers combine scores from generative models, such as phylogenetic evolutionary models and position-specific scoring matrices, whereas the classifiers used by CONTRAST take only the alignment itself as input. Finally, DOGFISH's global model is a generatively trained HMM, while CONTRAST uses a CRF trained to maximize expected coding region boundary accuracy.

In many ways, CONTRAST is less complex than other leading *de novo *gene predictors. Most predictors are based on a model that allows for semi-Markov dependencies between labels, such as a generalized HMM or a semi-Markov CRF; the fact that CONTRAST uses a standard CRF means its models of exon and intron lengths are fairly restricted. Moreover, CONTRAST does not explicitly model promoters, untranslated regions or conserved non-coding regions. Finally, the feature set used in CONTRAST is relatively simple: it contains no features designed to detect splicing branch points, polyadenylation signals or signal peptide sequences, for example. Incorporating more sophisticated biological models would likely improve predictive accuracy and is an important direction for future work. Other possible extensions to the system include adding the ability to predict untranslated regions, alternative splicing or overlapping genes.

## Conclusion

The failure of multiple alignment-based gene predictors to improve upon single informant performance in human has been a puzzling phenomenon. The situation runs counter to the strong intuitive feeling that additional genomes should provide enough extra statistical power to significantly increase predictive accuracy. At least one researcher was prompted to speculate that the lack of success could be a result of inadequate alignment quality or insufficient cross-species conservation of gene structure [30]. Our results suggest that the necessary information has been present in the alignments all along, but new methods were needed to effectively make use of it.

The greater precision of *de novo *gene prediction has two important consequences. First, better predictions should expedite efforts to verify the complete set of protein coding genes in human and other organisms experimentally. Targeted experiments designed to validate novel predictions will be able to recover more genes and operate at a higher efficiency than previously possible. Second, *de novo *predictions are becoming accurate enough that they can be relied upon with reasonable confidence. At present, testing computational predictions experimentally on a genome-wide scale is a time-consuming process. For many genomes, *de novo *predictions will provide an important annotation resource until annotation to a more exacting standard can be completed.

## Materials and methods

### Global model of gene structure

CONTRAST uses a CRF for its global model of gene structure. The CRF assigns a probability to each possible labeling **y **of a set of input data **x**. For our purposes, **x **consists of a genomic sequence from the target organism along with alignments from informant genomes and (optionally) ESTs, while **y **encodes a set of possible locations and structures of the genes in **x**.

**Figure 3:**
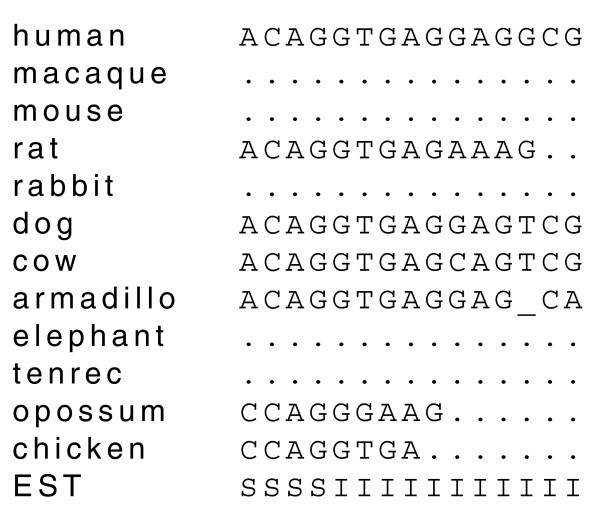
**Part of a typical set of input data**. The input data consists of 13 rows. The first row contains sequence from the target genome, the second to twelfth rows contain aligned sequence from informant genomes and the last row encodes information about the alignments of ESTs to the target genome.

Figure 3 shows part of a typical input to CONTRAST. The first row is the DNA sequence from the target genome. This row can contain characters corresponding to the four DNA bases as well as 'N'. Masked positions are indicated by lowercase letters. The next 11 rows are alignments from a variety of other species. These rows can contain the same characters as the first row, plus '_' for a gap in the alignment and '.' for unaligned positions. The final row contains a sequence which encodes information about EST alignments to the target genome. This row can contain five characters: 'N' for positions not covered by an EST alignment, 'S' for positions which all spliced EST alignments indicate to be in an exon, 'I' for positions which all spliced EST alignments indicate to be in an intron, 'C' for positions which some spliced EST alignments indicate to be an exon and some an intron and 'U' for positions covered by only unspliced EST alignments.

**Figure 4:**
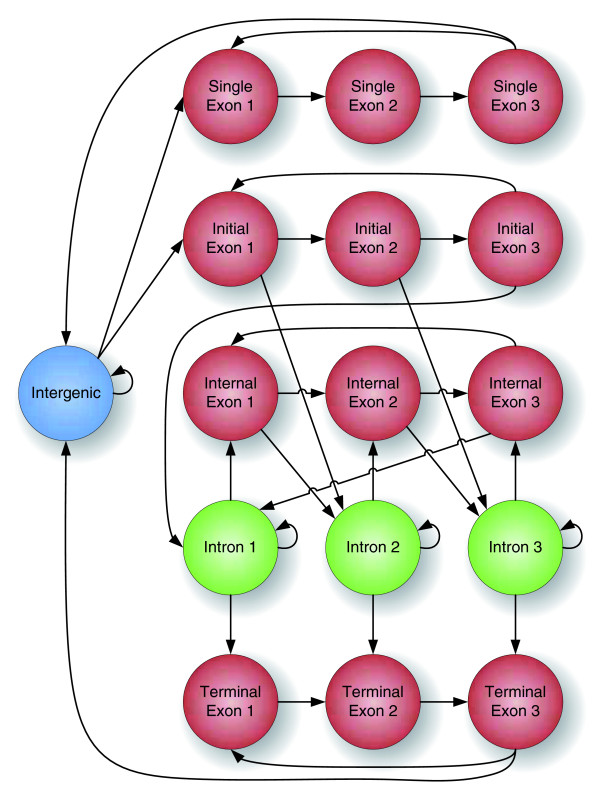
**The structure of labelings in CONTRAST**. Each node in the graph is a possible label for a single position in the target sequence. A labeling is legal if it corresponds to a path through the graph.

Figure 4 illustrates the structure of labelings in CONTRAST. Nodes in the diagram represent possible labels for a single position in the input data. Two labels are allowed to occur in succession only if there is an arrow from the first label to the second. Thus, a valid labeling corresponds to a path through the diagram. Labels for the coding sequence, shown as red nodes, are classified as either belonging to a gene with a single coding exon or to the initial, internal or terminal coding exon of a multi-exon gene. There are three labels (1, 2 and 3) for each exon type, corresponding to whether the position is the first, second or third base of a codon. Coding region boundaries correspond to neighboring labels of different colors. Each coding region boundary in the labeling must occur at a position with a valid consensus sequence in the target genome ('ATG' for start codons; 'TAA', 'TAG' or 'TGA' for stop codons; 'GT' or 'GC' for donor splice sites; and 'AG' for acceptor splice sites). The exon and intron labels in the diagram represent genes on one DNA strand only; the full model contains a symmetric set of labels (not shown) representing genes on the opposite strand. The full model also includes additional intron labels to track stop codons that are split across exons. For example, on the positive strand, the 'Intron 1' label is split into two labels, one for introns bordered on the 5*' *end by a coding 'T' and one for all other introns. Similarly, the 'Intron 2' label is split into three labels, depending on whether the previous two coding bases were 'TA', 'TG' or neither. These additional labels serve to disallow labelings containing genes with split in-frame stop codons.

The conditional probability of a labeling **y **given a sequence **x **is defined as

$$P(y|x)=\frac{e^{w^{T}F(x,y)}}{\sum_{y^{\prime }}e^{w^{T}F(x,y^{\prime })}},$$

where **w**, the weight vector, is learned from training data and **F**(**x**, **y**), the summed feature vector, is given by

$$F(x,y)=\sum_{i=1}^{L}f(y_{i-1},y_{i},i,x).$$

Here *L *is the length of the input data (and labeling), *y*_*i *_is the label at position *i *and **f**, called the feature mapping, is a vector-valued function which determines the information used to calculate the score of a position. The components of **f **are referred to as the CRF's features. For simplicity, we assume the existence of a special initial label *y*_0_.

Specifying **f **is the key task in designing a CRF. A few entries in the feature mapping for a simple CRF-based gene predictor are given as follows:

$$f(y_{i-1},y_{i},i,x)=\left[\begin{array}{c} 1\{y_{i-1}=Intron\;and\;y_{i}=Exon\}\\ 1\{y_{i}=Exon\;and\;x_{i}='G'\}\\ \cdots \end{array}\right].$$

Here 1{} is the indicator function, which returns 1 if its argument is true and 0 otherwise. In this example, a position receives a score of *w*_1 _if the label at the previous position is 'Intron' and the label at the current position is 'Exon', plus a score of *w*_2 _if the label at the current position is 'Exon' and the corresponding position in the sequence contains a 'G'. The score of a labeling, **w**^T^**F**(**x**, **y**), is simply the sum of the scores of all of its positions. The probability of a labeling is obtained by exponentiating its score and dividing by a normalizing constant which ensures that the probabilities of all labelings sum to one.

### Feature mapping

The feature mapping used in CONTRAST consists of three main types of features: features that score transitions between labels, features that score a label based on sequence near its position and features that score coding region boundaries.

Transition features are defined as follows. For each pair of labels *y *and *y' *such that *y *is allowed to follow *y'*, the feature mapping contains the following element:

**f**(*y*_*i*-1_, *y*_*i*_, *i*, **x**) = 1{*y*_*i*-1 _= *y' *and *y*_*i *_= *y*}.

**Figure 5:**
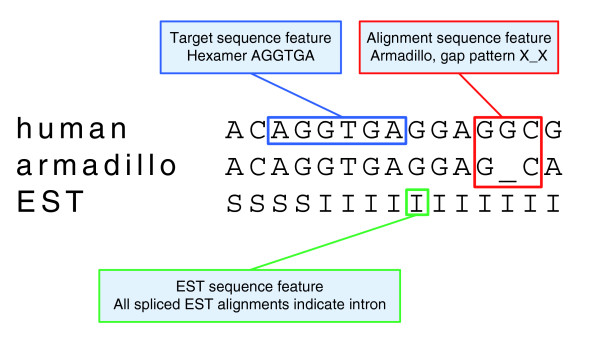
**Features that score a label based on local sequence**. CONTRAST contains three types of features for scoring a label based on local sequence: features based on hexamers in the target sequence (shown in blue), features based on a trimer in the target sequence and a trimer in an informant alignment (shown in red) and features based on a position in the EST sequence (shown in green).

There are three types of sequence-based features: features based on the target sequence, features based on the alignment of a particular species to the target sequence and features based on EST evidence. Figure 5 provides a graphical illustration of the three feature types, which we describe in detail in the following. Let *S*_intron _denote the set of candidate labels corresponding to intronic positions in a forward-strand gene of the target sequence and, similarly, let *S*_exon1_, *S*_exon2 _and *S*_exon3 _denote sets of labels corresponding to the three forward-strand coding frames, respectively. For example, *S*_exon1 _= {'Initial Exon 1', 'Internal Exon 1, 'Terminal Exon 1', 'Single Exon 1'}. We refer to each of these four label sets as forward strand label sets and define their reverse strand counterparts *Ŝ*_intron_, *Ŝ*_exon1_, *Ŝ*_exon2_, and *Ŝ*_exon3 _analogously.

For each DNA hexamer *h *and forward strand label set *S*, the feature mapping contains

$$1\{(y_{i}\in S\;and\;x_{1,i:i+5}=h)\;or\;(y_{i}\in \hat{S}\;and\;{\tilde{x}}_{1,i-5:i}=\tilde{h})\},$$

where *x*_*r*,*i*:*j *_are the characters in row *r *of the input data from positions *i *to *j *inclusive and $\tilde{h}$ is the reverse complement of *h*. Defining the features in this way ensures that a specific hexamer will be treated the same whether it is part of a gene on the forward or reverse strand. Additional features are included to score hexamers in the target sequence that contain an 'N':

1{(*y*_*i *_∈ *S *and *x*_1,*i*:*i*+5 _contains 'N') or (*y*_*i *_∈ *Ŝ *and *x*_1,*i*-5:*i *_contains 'N'}.

Finally, masked positions, which are indicated in the target sequence by lowercase characters, are scored using a set of features of the form:

1{(*y*_*i *_∈ *S *or *y*_*i *_∈ *Ŝ*) and *x*_*i *_is lowercase}.

Thus, each label (other than 'Intergenic') has 4,098 target sequence features associated with it: one for each possible hexamer consisting of only DNA bases, one for all hexamers containing an 'N' and one for masked positions.

Alignment sequence features are based on the combination of a trimer in the target sequence and a trimer in one of the aligned sequences. For each forward strand label set *S*, row *r *of the input data corresponding to an informant alignment and pair of DNA trimers *t *and *t'*, the feature mapping contains

$$1\{(y_{i}\in S\;and\;x_{1,i:i+2}=t\;and\;x_{r,i:i+2}=t^{\prime })\;or\;(y_{i}\in \hat{S}\;and\;{\tilde{x}}_{1,i-2:i}=\tilde{t}\;and\;{\tilde{x}}_{r,i-2:i}={\tilde{t}}^{\prime })\}.$$

For aligned trimers containing gaps but no unaligned characters, the feature mapping takes into account only the configuration of gaps in the trimer and ignores any DNA base characters. This is accomplished through use of the concept of a gap pattern. A gap pattern is a string consisting of the characters '_' and 'X'. A string *s *matches a gap pattern *g *if *s *contains a '_' character at every position that *g *does and a DNA base character at every position that *g *has an 'X'. For example, the strings 'A_G', 'C_T' and 'A_A' all match the gap pattern 'X_X'. For each forward strand label set *S*, alignment row *r *and possible three-character gap pattern *g *except 'XXX', the feature mapping contains

$$1\{(y_{i}\in S\;and\;x_{r,i:i+2}\;matches\;g)\;or\;(y_{i}\in \hat{S}\;and\;x_{r,i-2:i}^{R}\;matches\;g^{R})\},$$

where *s*^R ^indicates the reversal of string *s*. Finally, trimers containing unaligned characters are divided into three groups: those representing the start of an alignment ('..X' and '.XX'), those representing the end of an alignment ('X..' and 'XX.') and those that are completely unaligned ('...'). Here, 'X' is interpreted as meaning any character other than '.'. For each such group *g*, forward strand label set *S *and alignment row *r*, the feature mapping contains

$$1\{(y_{i}\in S\;and\;x_{r,i:i+2}\;\in \;g)\;or\;(y_{i}\in \hat{S}\;and\;x_{r,i-2:i}^{R}\;\in \;g^{R})\}.$$

EST sequence features are the most straightforward. For each forward strand label set *S *and EST sequence character *e*, the feature mapping contains

1{(*y*_*i *_∈ *S *or *y*_*i *_∈ *Ŝ*) and *x*_est,*i *_= *e*},

where *x*_est,*i *_is the EST sequence character at position *i*.

Features for scoring coding region boundaries using alignment information are based on outputs from a set of binary classifiers. Each boundary type (start codon, stop codon, GT donor splice, GC donor splice and acceptor splice) has an associated classifier. The classifier takes as input a small window around two neighboring positions in the alignment potentially corresponding to a boundary and outputs a decision value. Large positive decision values indicate a confident prediction that the positions in question do in fact constitute a boundary of the classifier's type, highly negative values indicate a confident negative prediction and values near zero indicate an uncertain prediction. We chose not to directly use the classifier's decision value as a feature, because this would result in the score of a boundary being a simple linear function of the classifier's output. To allow for a more general relationship, CONTRAST maps the output of a classifier to a score using a piecewise linear function interpolated through a set of control points. More precisely, the score of a decision value *d *is given by

$$\begin{array}{c} s(d)=(1-c)w_{i}+cw_{i+1},\\ c=\frac{d-x_{i}}{x_{i+1}-x_{i}}, \end{array}$$

where (*x*_*i*_, *w*_*i*_) and (*x*_*i*+1_, *w*_*i*+1_) are control points selected such that *d *∈ [*x*_*i*_, *x*_*i*+1_] if possible or the two points with *x*-coordinates closest to *d *if not. The number of control points and their *x*-coordinates are fixed before CRF training (see the training procedure section), while the *w*-coordinates of the control points are learned by the CRF (that is, they correspond to components of the weight vector **w**). This is accomplished by specifying the feature mapping such that it contains one feature for each control point (*x*_*i*_, *w*_*i*_) associated with a particular classifier. Each such feature has non-zero value only if the labels (*y*_*i*-1_, *y*_*i*_) correspond to a boundary of the classifier's type and the classifier's score at position *i *is such that (*x*_*i*_, *w*_*i*_) is used as a control point to determine the CRF score. In that case, the feature's value is equal to the interpolation coefficient of its associated control point: (1 - *c*) if it is the control point with the lower *x*-coordinate and *c *otherwise.

CONTRAST also includes features that score coding region boundaries based on EST sequence. For each coding region boundary type *b *and pair of EST sequence characters *e *and *e'*, the feature mapping contains

1{(*y*_*i*-1_, *y*_*i*_) corresponds to *b*, *x*_est,c _= *e *and *x*_est,nc _= *e'*}.

**Figure 6:**
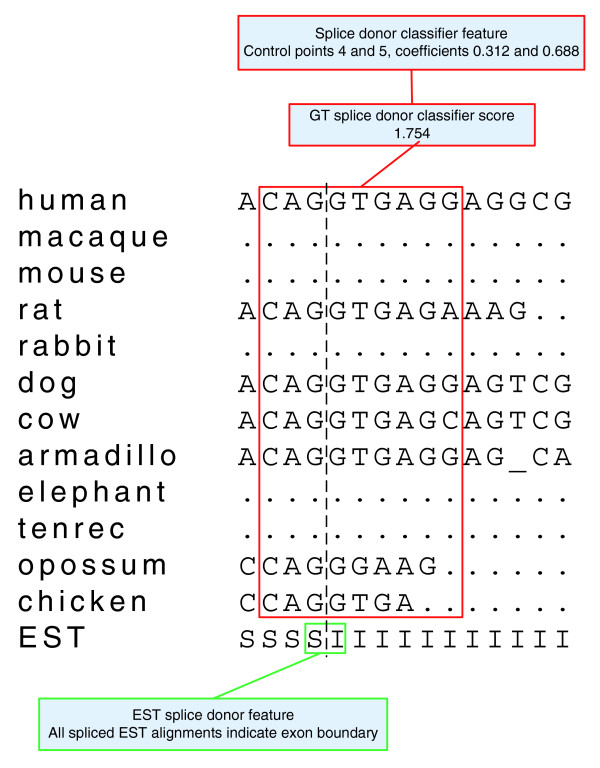
**Features that score coding region boundaries**. CONTRAST contains two types of feature for scoring coding region boundaries. The first, shown in red, maps the output of a classifier to a score using a piecewise linear function learned during CRF training. In this example, the score from the GT splice donor classifier falls between the fourth and fifth control points for the function, with interpolation coefficients of 0.312 and 0.688. The second type of feature, shown in green, scores a coding region boundary based on the EST sequence characters that flank it.

Here, *x*_est,c _is the EST sequence character at the coding position closest to the boundary, while *x*_est,nc _is the EST sequence character at the non-coding position closest to the boundary. Figure 6 provides an illustration of the two types of feature that score coding region boundaries.

### Coding region boundary classifiers

CONTRAST uses SVMs as its coding region boundary classifiers. We use a set of binary features to define a one-to-one-mapping from the space of multiple alignment windows to the space of SVM input vectors. For each possible character *c*, row *i *and column *j *in the window, the SVM feature vector contains an element

1{the character in row *i *and column *j *is *c*}.

We use quadratic kernels for the SVMs, allowing them to operate implicitly in a much larger feature space. For each pair of features *f*_*i *_and *f*_*j *_in the original space, the expanded feature space contains the feature *f*_*i*_*f*_*j*_. As *f*_*i *_and *f*_*j *_are binary features, *f*_*i*_*f*_*j *_is also a binary feature whose argument is the conjunction of the arguments to *f*_*i*_and *f*_*j*_. An example feature in the expanded space is

1{the character in position 4 of the 'mouse' row is 'G' and the character in position 5 of the 'rat' row is 'T'}.

Table 6 lists the window sizes and positions for the five boundary classifiers used in CONTRAST, along with the position of a required consensus sequence. The coordinates in the table are defined such that 1 is the position immediately 3' of the coding region boundary, *-*1 is the position immediately 5' of the boundary and coordinates increase in the 5' to 3' direction.

**Table 6:** Coding region boundary classifiers. Coding region boundary classifiers. Window sizes and positions are shown for the five coding region boundary classifiers used by CONTRAST. Coordinates are defined such that the boundary occurs between the adjacent positions -1 and 1 (that is, either position -1 is coding and position 1 is coding or the reverse is true), with coordinates increasing in the 5' to 3' direction. Each classifier's require consensus sequence is shown in the second column.

	Consensus	5' end	3' end	Length
Start codon	A_1_T_2_G_3_	-8	6	14
Stop codon	T_1_A_2_A_3_, T_1_A_2_G_3_, T_1_G_2_A_3_	1	6	6
Donor splice GT	G_1_T_2_	-3	8	11
Donor splice GC	G_1_C_2_	-3	8	11
Acceptor splice	A_-2_G_-1_	-27	3	30

### Maximum boundary accuracy decoding

The standard procedure for using a CRF to predict labels for a new sequence involves selecting the labeling with the maximum conditional likelihood given the sequence,

$$y=\underset{y^{\prime }}{\arg\max}P(y^{\prime }|x).$$

Instead, we select the labeling that maximizes a weighted difference between the expected number of true-positive and false-positive coding region boundary predictions:

$$\begin{array}{c} y=\underset{y^{\prime }}{\arg\max}\sum_{j=2}^{L}1\{({y^{\prime }}_{j-1},{y^{\prime }}_{j})\in B\}A({y^{\prime }}_{j-1},{y^{\prime }}_{j}),\\ A({y^{\prime }}_{j-1},{y^{\prime }}_{j})=\kappa P({y^{\prime }}_{j-1},{y^{\prime }}_{j}|x)-(1-P({y^{\prime }}_{j-1},{y^{\prime }}_{j}|x)). \end{array}$$

Here, *B *is the set of all pairs of labels corresponding to a coding boundary and *κ *is a constant that controls a tradeoff between sensitivity and specificity. We used *κ *= 1 for the human experiments.

For the *Drosophila melanogaster *experiments, we chose *κ *= 1.5 after observing that this higher value of *κ *led to slightly better performance.

We can efficiently compute **y **by first running the forward and backward algorithms to calculate the posterior probabilities of all pairs of adjacent labels, and then running a Viterbi-like dynamic programming algorithm to find the optimal labeling.

### Maximum expected boundary accuracy training

Standard algorithms for training a CRF are based on maximum conditional likelihood. Given a set of training examples $D={\left\{x^{\left(t\right)},y^{\left(t\right)}\right\}}_{t=1}^{m}$, conditional likelihood training finds weights for the CRF that maximize the conditional likelihood of the training data,

$$L(w)=\prod_{t=1}^{m}P(y^{\left(t\right)}|x^{\left(t\right)}).$$

Instead, we optimize the expected boundary accuracy on the training set, which we define as

$$\begin{array}{c} EBA(w)=\sum_{t=1}^{m}\sum_{j=2}^{L_{t}}A_{j}^{\left(t\right)},\\ A_{j}^{\left(t\right)}=1\{(y_{j-1}^{\left(t\right)},y_{j}^{\left(t\right)})\in B\}\lambda P(y_{j-1}^{\left(t\right)},y_{j}^{\left(t\right)}|x^{\left(t\right)})-\sum_{\left({y^{\prime }}_{j-1},{y^{\prime }}_{j})\in B-(y_{j-1}^{\left(t\right)},y_{j}^{\left(t\right)}\right)}P({y^{\prime }}_{j-1},{y^{\prime }}_{j}|x^{\left(t\right)}). \end{array}$$

Informally, EBA(**w**) is the weighted sum of the probabilities of all correct coding boundary labels and the negative probabilities of all possible incorrect coding boundary labels. Note that the training data may contain coding boundaries from unannotated genes, which will be penalized as incorrect. In such cases, a relatively high value of *λ *may be required to offset spurious penalties. We used *λ *= 15 for all experiments.

The choice to use maximum expected boundary accuracy training for CONTRAST was motivated by a previous result demonstrating that a training criterion based on maximum accuracy led to far better performance than maximum conditional likelihood training for a gene prediction task [44].

In practice, we optimize EBA(**w**) using a gradient-based optimization algorithm (described in the following). The gradient can be calculated efficiently using a dynamic programming algorithm that is only a small constant factor slower than the algorithm used to compute the gradient of *L*(**w**). The techniques used are very similar to those described previously [44]; we omit the details for reasons of brevity.

### Cross-validation procedure

The cross-validation procedure we used was designed to estimate CONTRAST's ability to discover unannotated genes when trained on and used to generate predictions for an entire genome. We divided the annotations of known genes into four sets at random. At each iteration of the cross-validation procedure, we trained CONTRAST using only annotations from three of the four sets. We then generated predictions for the full genome and counted the number of correctly predicted nucleotides, exons and genes not included in the training set. The results for each iteration were summed to calculate the total number of correct predictions. To determine specificity, we considered the number of predictions made by CONTRAST to be the average of the number of predictions made at each iteration.

### Training procedure

To train CONTRAST, we first split the target genome into fragments of length 1 Mbp. The fragments were then randomly divided into three sets: 25% went into a set used for SVM training, 50% went into a set used to train the CRF and the remaining 25% formed a holdout set used to estimate generalization accuracy.

As CONTRAST predicts only one transcript per gene, we used only one randomly selected transcript of each gene for training.

For each example of a particular type of coding region boundary that occurs in the SVM training set, we created a positive training example for the appropriate SVM. We also created a negative example by searching, in a randomly selected direction, for the nearest occurrence of the SVM's required consensus in the target sequence. We chose this method of selecting negative examples over simply sampling uniformly from the target genome for two reasons. First, negative examples taken from positions near a true coding boundary display a relatively high level of conservation, making them difficult to classify. Second, these negative examples correspond to plausible mispredictions in which a coding boundary is predicted slightly 5' or 3' of its true location. We also extracted a second set of positive and negative examples by applying the above procedure to the CRF training set. This set was reserved for estimating the generalization performance and was not used directly in SVM training. After the SVM examples were constructed, we trained standard two-class SVMs using code from the libsvm library [45]. The slack variable coefficient *C *was selected to maximize classification accuracy on the examples from the CRF training set.

After SVM training was complete, we set the abscissas of the control points used to map SVM decision values to CRF scores. For each SVM, the outermost two abscissas were selected such that they bracketed 99% of the decision values for positive examples in the CRF training set. The remaining abscissas were placed at uniform intervals between those two. Ten control points were used for the GT donor splice and acceptor splice classifiers; the other classifiers used five control points. An initial CRF weight vector **w **was then computed as follows. Weights for transition features were set according to the formula

$$w_{ij}=log\left(\frac{N_{ij}}{N_{i}}\right).$$

Here *w*_*ij *_is the weight for a transition from label *i *to label *j*, *N*_*i *_is the number of times label *i *appeared in the CRF training set and *N*_*ij *_is the number of times label *j *appeared immediately after label *i *in the CRF training set. Weights corresponding to the ordinates of control points were set according to the formula

$$w_{i}=log\left(\frac{P_{i}}{N_{i}}\right).$$

Here *w*_*i *_is the ordinate of control point *i*, *P*_*i *_is the number of positive examples in the CRF training set with a decision value such that *i *would be used to compute its CRF score and *N*_*i *_is the corresponding number for negative examples. The remaining weights were set to small random values chosen uniformly from the interval [-10^-3^, 10^-3^]. The process of selecting an initial weight vector should not be interpreted as a parameter estimation procedure, as the final CRF weights bear little resemblance to their initial values. Rather, the above procedure serves to initialize the CRF learning algorithm at a reasonable starting point.

Finally, the weight vector was optimized to maximize the function

$$EBA(w)-C\sum_{i}w_{i}^{2}.$$

Here the first term is the expected boundary accuracy for the CRF training set (explained previously), while the second is a regularization term added to combat overfitting.

The sum in the regularization term is over weights for hexamer and trimer pair features only. The other weights were not subject to regularization, because their associated features occurred often enough in the training data that overfitting was not a significant problem.

The Rprop algorithm was used for optimization [46]. We did not run Rprop to convergence, but rather applied an early stopping procedure: optimization was terminated if five iterations occurred without an improvement in the value of the expected boundary accuracy for the holdout set. The regularization constant *C *was selected by performing a golden section search [47] to determine which value led to the best expected boundary accuracy for the holdout set. Training CONTRAST on the human genome with 11 informants required approximately 12 hours using 200 Intel Xeon E5345 processors (2.33 GHz) in parallel.
